# Research on the Relationship between Physical Literacy, Physical Activity and Sedentary Behavior

**DOI:** 10.3390/ijerph192416455

**Published:** 2022-12-08

**Authors:** Wenjing Yan, Yihan Meng, Lina Wang, Ting Zhang, Leqin Chen, Hongjuan Li

**Affiliations:** 1 Key Laboratory of the Ministry of Education of Exercise and Physical Fitness, School of Sport Science, Beijing Sport University, Beijing 100084, China; 2 School of Physical Education, Shanxi Normal University, Taiyuan 030000, China

**Keywords:** physical literacy, physical activity, sedentary behavior

## Abstract

During the COVID-19 pandemic, college students’ health-related physical activity and physical literacy aroused widespread concern. This study evaluated the relationship among physical literacy (PL), sedentary behavior (SB), light physical activity (LPA), and moderate-to-vigorous physical activity (MVPA); we further explored whether LAP and SB mediated the association between PL and MVPA. Methods: This study was based on a cross-sectional survey of Chinese college students. The Perceived Physical Literacy Instrument Scale (PPLI-SC) and International Physical Activity Questionnaire Short Form (IPAQ-SF) were used to investigate the PL, MVPA, LPA, and SB. Results: There were 2996 valid questionnaires with 829 boys and 2167 girls. The MVPA, LPA, and PL of boys were significantly higher than girls, while the SB values were significantly lower in girls (*p <* 0.01). The correlation analysis showed that there was a significant correlation between the two indexes except for SB and LPA. Path analysis shows that PL directly, significantly, and positively affects MVPA. PL reduces SB (β = −0.085, *p* < 0.001) and increases LPA (β = 0.097, *p* < 0.001). The total mediation effect accounted for 14.014%, and the mediation effects of SB and LPA accounted for 4.417% and 9.597%, respectively. Conclusions: LPA and SB partially mediated the relation between PL and MVPA. SB and LPA partially explain the impact of PL on MVPA. The findings suggest that managing SB and improving LPA could play a significant indirect role in increasing the positive effect of PL on MVPA and that increasing the opportunities for LPA increased the MVPA for Chinese college students.

## 1. Introduction

Regular physical activity (PA) can produce a series of health benefits and reduce the disease incidence rate and mortality [1]. A lack of PA endangers mental health and reduces quality of life [2]. The 2020 WHO guidelines recommended that adults should have 150–300 min of moderate intensity physical activity, or 75–150 min of high-intensity physical activity (or the equivalent combination of moderate intensity and high-intensity aerobic physical activity) every week [3]. Versus the total weekly MVPA accumulation, the frequency of moderate-to-vigorous physical activity (MVPA) is more important for health promotion [4]. The health benefits of light physical activity (LPA) are similar to MVPA and indicate better physical health and performance. This insight suggests that LPA and MVPA are equally important. With increasing sedentary behavior (SB), college students’ stress, anxiety, and depression have increased significantly [5,6]. The COVID-19 pandemic has led to a significant increase in SB and a significant decrease in the PA level of college students [7,8]. Students have not participated in PA to offset the increase in SB [9]. Concurrently, obesity has become a major public health problem in China. The prevalence of overweight and obesity has increased rapidly in the past 40 years. More than 34.3% of adults are overweight, with an obesity rate of 16.4% [10]. However, college students’ dietary patterns, physical activity, as well as sedentary and other unhealthy lifestyle behaviors have coexisted and interacted with each other, thus increasing the risk of being overweight and obese, and being obese leads to a further decline in physical activity level [11]. There is also a general lack of awareness about obesity among college students, which increases the risk of obesity [12]. Clearly, a lack of exercise and obesity are major public health challenges.

Higher education is a very important stage in an individual’s life in China. The individual’s education mode has changed from compulsory physical education in junior and middle school to a more independent adult physical lifestyle. The stage of higher education is a period when students’ physical activity level changes significantly [13]. Short-term health interventions have had a positive impact on a variety of health behaviors among college students [14]. Recently, many countries have begun to develop intervention models based on physical literacy to improve physical activity and health [15]. Physical literacy (PL) refers to the ability to be competent and confident in various sports activities across a variety of environments, and is conducive to the healthy development of the whole person, including emotion, body, and cognition [16]. PL is a multi-disciplinary and comprehensive concept. It is a prerequisite for individuals to participate in and adhere to sports activities throughout their life, and lays a foundation for individuals’ ability and tendency to engage in lifelong physical activities [17]. PL can improve the quality and quantity of participation in sport and physical activity throughout life [18]. It is also necessary to develop a physically literate population, who meaningfully engage in play and physical activity throughout the development of functional movement skills in enriched environments. PL and PA levels were significantly related [19]. People who are physically inactive generally had lower PL scores [20]. Many studies have discussed the extension and application of PL in the area of cross-sectional study [15,21]. These studies show that the attributes of PL are concentrated mainly in motivation, physical competency and knowledge [21]. Physical literacy is also an important factor affecting physical activities [19]. However, our understanding of the relationship between PL, MVPA, LPA and SB are limited, and we are unaware of relevant prior research. Thus, our goal here was to explore the relationship between PL and MVPA, LPA, and SB. We further provide a theoretical reference for formulating more effective health interventions and obesity-intervention measures. This study consists of two parts: (1) investigating the status of PL and MVPA, LPA, and SB of college students using a survey; (2) exploring the relationship between PL, MVPA, LPA, and SB.

## 2. Materials and Methods

### 2.1. Study Design and Participants

This was a single-center cross-sectional study from May to June 2022. College students were recruited to complete questionnaires in China. The teachers distributed the questionnaire to students to fill in voluntarily. Students’ PL, SB, LPA and MVPA were measured online via a Perceived Physical literacy Scale (PPLI-SC) [21] and the International Physical Activity Questionnaire (IPAQ-S) [22]. Demographic information (age, sex, specialty, height, and weight) was collected, and body mass index (BMI) was calculated using self-reported weight and height.

Participants were randomly recruited to fill in electronic questionnaires. All patients provided informed consent. After removing invalid questionnaires, 2996 valid questionnaires remained. Before completing the questionnaire, the content of the study was fully explained, and all participants gave informed consent, indicating that he or she voluntarily participated in the study. This study followed the Declaration of Helsinki and was approved by the Scientific Experiment Ethics Committee of Beijing Sport University (2019101 H).

### 2.2. Physical Literacy

Participants’ perceived PL was assessed by PPLI-SC translated from PPLI. This is an eight-item instrument used to measure the perceived PL of Chinese undergraduates [21]. It includes three dimensions: (1) motivation, (2) confidence and physical competence, and (3) interaction with the environment. These dimensions are core attributes of Whitehead’s PL concept [17]. The scale used a five-point Likert scale, with responses ranging from strongly disagree to strongly agree on a scale of 1–5. The PL result was summed to give an overall score between 8 and 40, with higher scores indicating better physical literacy.

### 2.3. Physical Activity

Physical activity levels were measured using the IPAQ-SF. At least 10 min of uninterrupted PA performance were recorded for one week (five school days and two weekend days). There were seven questions: six focusing on physical activity, and one focused on sedentary behavior. The metabolic equivalent time (MET) of LPA was 3.3, and the MET of MPA was 4.0. VPA was 8.0. Data cleaning and outlier processing was performed if the physical activity of a certain intensity exceeded three hours per day. Then, it was re-coded as 180 min. If the report was less than 10 min, then we recorded “0”. The individual’s weekly physical activity level was based on the number of minutes of MET value, calculated using the MET-minutes calculation formula. That is, the number of minutes of physical activity corresponding to the MET value multiplied by the weekly frequency (D/W). The MVPA level was the sum of MPA and LPA [22].

### 2.4. Mediating Effect

The independent variable X had an impact on the dependent variable Y. If X affected Y by influencing variable M in addition to directly influencing Y, then M was called the intermediate variable. This was called the mediation effect. In essence, there were four regression equations for parallel mediations as shown below. The independent variable X was PL, the intermediary variable M_1_ was SB, the intermediary variable M_2_ was LPA, and the dependent variable Y was MVPA. Term c represents the total effect of X on Y. Term a represents the effect of X on M, c’ represents the effect of X on Y after controlling M, and b represents the effect of M on Y after controlling X.

Y = i + cX + e(1)

M_1_ = i_M1_ + a_1_X + e_M1_ (2)

M_2_ = i_M2_ + a_2_X + e_M2_ (3)

Y = i_Y_ + c’X + b_1_M_1_ + b_2_M_2_ + e_Y_ (4)

### 2.5. Data Analysis

IBM SPSS 22 was used for data analysis. Model 4 in the process 4.0 plug-in was used to test the mediation effect. Descriptive statistical analysis of demographic indicators was conducted, and the independent sample T test was used to compare gender differences. The coefficient of variation was studied for variability analysis. Pearson correlations and linear regression fitting in regression analysis analyzed the relationship between each index at α = 0.05.

## 3. Results

### 3.1. Characteristics of Students

Descriptive statistics showed that there were 2996 subjects including 829 boys, accounting for 27.7% and 2167 girls, accounting for 72.3%. The average age was 20.16 ± 1.21 years old (Table 1).

### 3.2. Gender Difference

The results showed that the scores of MVPA, LPA and PL of boys were significantly higher than girls (*p* < 0.01). Those of SB girls were significantly higher than boys (*p* < 0.01). The variation degree of MVPA and LPA of girls was greater than SB and PL of boys (Table 2).

### 3.3. Variable Differences of Different Weight Status Categories

**Figure 1:**
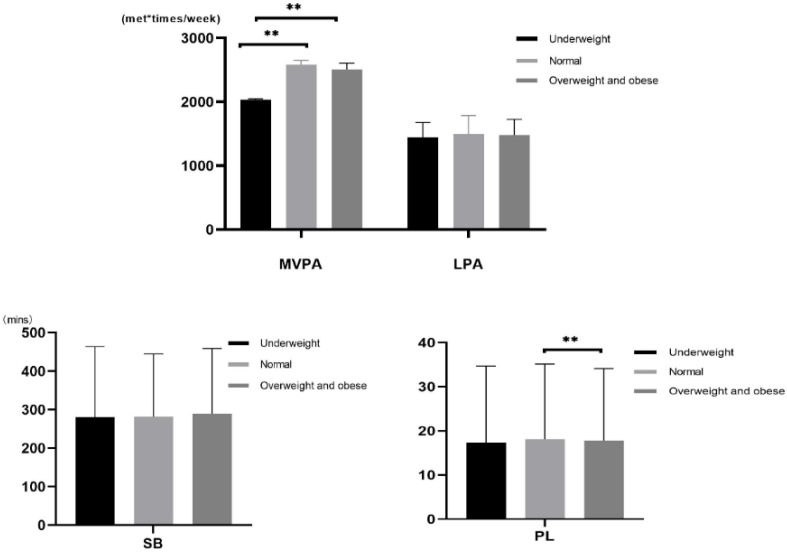
Multiple Comparisons (** *p* < 0.01).

The population mean of MVPA and PL at different obesity levels was different, while the population mean of SB and LPA was not different (Table 3). Here, the MVPA of normal weight and obesity was significantly higher than underweight subjects. The PL of normal weight was significantly higher than obese subjects (Figure 1).

### 3.4. Correlation Analysis of Variables

The correlation coefficient between PL and MVPA was 0.316 (*p* < 0.01). PL was positively correlated with LPA, MVPA, and negatively correlated with SB (*p* < 0.01). There was no correlation between SB and LPA (*p* > 0.01). The correlations, although significant, were not strong (Table 4).

### 3.5. Regression Analysis and Intermediary Effect Test

**Figure 2:**
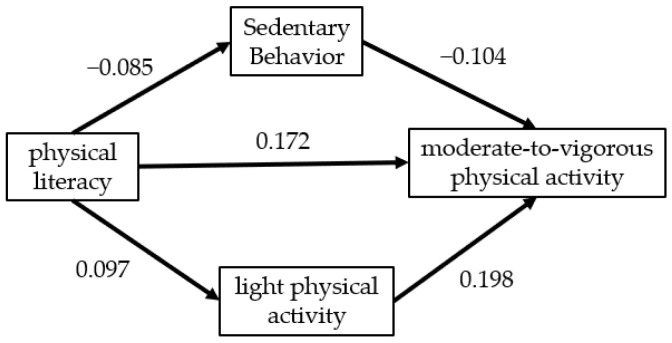
Mediation model diagram of SB and LPA.

According to the correlation between the research variables, a parallel mediation model was constructed to explore the mechanism of SB and LPA in PL and MVPA (Figure 2).

Table 5 shows that the four regression equations were constructed by parallel mediation effect analysis to test the mediation effect of SB and LPA in the relationship between PL and MVPA under the control of gender, grade and major. Model 2 showed that the direct effect of PL on MVPA was still statistically significant after the intermediary variables SB and LPA were included (β= 0.172, *p* < 0.001); SB was negatively correlated with MVPA after PL control (β = −0.104, *p* < 0.001); PL had a negative effect on SB (β = −0.085, *p* < 0.001); LPA was positively correlated with MVPA (β = 0.198, *p* < 0.001); and PL had a positive effect on LPA (β = 0.097, *p* < 0.001). The results of regression analysis showed that the regression equations before and after model adjustment were statistically significant, and the coefficients c, c’, a, and b were significant. This indicates that SB and LPA partially mediated between PL and MVPA.

The bootstrap method was used to repeatedly (5000 times) sample and test the mediation effect. The 95% confidence interval of the mediating effect of path 1 and path 2 did not include 0, thus indicating that the mediating effect was statistically significant. The total mediating effect accounts for 14.014%, and the mediating effect of path 1 and path 2 accounts for 4.417% and 9.597% of the total effect, respectively (Table 6).

## 4. Discussion

To the best of our knowledge, this is the first study where LPA and SB were parallel multiple mediators of the relationship between PL and MVPA among Chinese university students. The results showed that there was a significant correlation between the other indexes except SB and LPA. PL significantly and positively affects MVPA, similar to previous results [19,20]. We found that LPA and SB partially mediated the relationship between PL and MVPA. The SB and LPA terms could partially explain the impact of PL on MVPA. Part of the impact of PL on MVPA was direct, and the other part was realized by influencing SB and LPA. Our research underscores the fact that subjects needed not only to improve their PL but also to manage SB and increase LPA in the measures to promote active MVPA. We also found that boys’ PL, MVPA and LPA were significantly higher than girls’; boys’ SB was significantly lower than girls’. Boys’ MVPA and LPA had greater variability, while girls’ SB and PL had greater variability.

A growing number of countries use the theory of physical literacy to improve physical activity. The college education stage is an important time period for students to accept basic knowledge of PL and actively participate in physical activities. PL-based interventions could effectively reduce the PA decline observed in college students during the first year of school, while also helping to maintain students’ physical health [23]. The Ministry of Education of China requires Chinese colleges to provide no less than 144 h of compulsory physical education courses for freshmen and sophomores. Physical literacy came from the field of sports and emphasizes the importance of forming a lifelong exercise habit. Cairney et al. [24] proposed PL-based intervention as a framework model for improving PA and physical health. They positioned both PA and PL as models of health determinants and conducted empirical research and discussion. Miller et al. [25] conducted a mixed physical literacy intervention in Hong Kong students via a “standing and moving” randomized controlled trial with protocol and baseline characteristics. The results showed that the classroom interventions improved children’s health behaviors and were supported by school stakeholders and children’s families (i.e., parents). Celeste et al. [26] reported that participation in purposeful, social, and diversified activities, understanding age-related changes; and being able to actively adapt to changes were the foundations of becoming adults with good physical fitness. The stage of higher education was the last chance for students to accept the basic knowledge of PL and have a positive attitude towards sports activities. The comprehensive school physical activity plan currently implemented in the United States is based on physical literacy [27]. The purpose of the program was to enable students to acquire knowledge and skills related to the formation of physical literacy, enhance students’ willingness and motivation to participate in physical exercise, and cultivate students to form the habit of lifelong exercise. The environmental context in which PL projects were implemented is important—interventions to develop PL could only be effective in an interesting and enjoyable environment. If individuals felt unhappy, then it would be difficult for them to gain the confidence and motivation to participate in sports activities [24].

Therefore, intervention measures in such a special period of higher education is critical. Such interventions might lead to an active and healthy lifestyle during participation and could assist in the transition from high school to college. Our results showed that PL-based physical activity is very important to improve students’ health. PL provides a new framework for physical activity and health-promotion interventions that consider life-long physical activity engagement strategies across the lifespan.

There were differences in MVPA and PL among different levels of obesity, but no difference in SB. MVPA levels in the normal weight group were higher than in the overweight and obese group, but there was no significant difference. Similar results were obtained in Denmark [20], Spain [28] and Canada [29]. Relevant studies have found that obese children have fewer daily activities related to physical activity than non-obese children [30]. Studies have shown that the fear of exercise due to pain in obese people may limit their participation in health-promotion behaviors, thus resulting in lower levels of physical activity [31]. Studies have investigated the role of different sources of social support in the relationship between BMI and PA and found that family support and teacher support moderate on PA in overweight and obese adolescents. Peer support, relationships with peers, and social support can help adolescents proactively obtain more PA, especially obese adolescents [32]. We found that obese and underweight students had lower levels of physical activity. The PL of overweight and obese students is also significantly lower than normal weight students. Studies have found that psychological stress is related to the lower enjoyment of physical activities among overweight or obese adolescents [33]. The PL scores of obese children were significantly lower than normal weight children. Body composition variables were negatively correlated with PL (r from −0.223 to −0.507) [28]. Caldwell and colleagues [34] reported a negative association between body fat percentage and PL, health-related quality of life, and blood pressure using adolescent Physical Literacy Assessment (PLAY). The sports barriers of the young people after 16 years old came from the traditional physical education curriculum giving priority to sporting ability [35]. Therefore, a shift toward inclusive pedagogical models with an emphasis on a holistic approach might best promote the physical literacy necessary for the competence and confidence to continue movement in a lifelong capacity. Youth physical education is more important. If you did not want to go to college due to being overweight, you must start as soon as possible.

Little research has been conducted on the lifestyle and physiology of underweight people, the correlation between metabolism and weight loss, and genetics. Some studies report that high physical activity levels or a lack of exercise are associated with thermogenesis [36]. Others found that underweight people had more SB and less MVPA, leading to their lower overall PA. [37] Asian adolescents with lower BMI also had lower MVPA than adolescents with normal BMI [38]. The results of this study found that the MVPA of underweight people was significantly lower than that of normal and obese people with a higher SB. There were a few factors that affected the physical activity level of underweight people, and these should be further explored.

Studies have shown that the health benefits produced by LPA were similar to MVPA and were related to indicators of physical health [39], thus indicating that LPA and MVPA were equally important. Versus short-term MVPA, LPA maintained for a long time throughout the day is more beneficial to reduce inflammation and improve insulin resistance [40]. Some studies have found that replacing sedentary behavior with mild physical activity helped to maintain the cognitive function of elderly men. The results highlight the importance of behavior change in promoting cognition [41]. Participants met the recommendations of the daily MVPA, but they still had a high SB daily behavior pattern. In this case, LPA is an effective alternative to reduce SB and improve the health indicators of the population [42]. These studies support the selection of an appropriate, targeted and more feasible health promotion approach to achieve beneficial lifestyle changes. Increasing LPA may also be a potential lifestyle intervention to improve health [43]. The 2020 WHO guidelines reaffirmed messages that some physical activity is better than none. More physical activity is better for optimal health outcomes and provides a new recommendation on reducing sedentary behavior [3]. The findings contributed to the evidence that managing SB and improving LPA could play a significant indirect role in increasing the positive effect of PL on MVPA. Increasing the opportunities for LPA is more conducive to increasing MVPA for Chinese college students. The findings provided new evidence on the associations between physical activities and quality of life in college students.

This study does have some limitations. First, the PA and PL were self-reported, which is limited by recall bias and overestimation. Second, this was a cross-sectional study, and the sample consists of college students living in the north with a larger proportion of males than females, which might cause some deviation of results. As such, any generalization should be made cautiously.

## 5. Conclusions

We found that LPA and SB partially mediated the relation between PL and MVPA. SB and LPA could partially explain the impact of PL on MVPA. Our research highlighted the fact that in the measures to promote active MVPA, subjects not only needed to improve their PL but also needed to manage SB and increase LPA. The findings suggest that managing SB and improving LPA could play a significant indirect role in increasing the positive effect of PL on MVPA. Increasing the opportunities for LPA was conducive to increasing MVPA for Chinese college students. Therefore, we should identify students’ SB and LPA before establishing strategies to increase PL and to promote PA. We should also focus on girls and underweight people. More research is also needed to develop appropriate and tailored interventions focusing on these mediators.

## Figures and Tables

**Table 1:** Characteristics of students.

	Boy N = 829 (27.7%)	Girl N = 2167 (72.3%)	All (2996)
Age (years)	20.33 ± 1.22	20.09 ± 1.20	20.16 ± 1.21
High (cm)	177.64 ± 6.07	163.24 ± 5.20	167.23 ± 8.44
Weigh (kg)	71.48 ± 12.77	54.67 ± 7.98	59.32 ± 12.16
Bmi (kg/m^2^)	22.61 ± 3.57	20.50 ± 2.72	21.08 ± 3.13
Grade			
1	1425	47.50%	
2	725	24.19%	
3	846	28.23%	
Major			
PE	458	15.30%	
Non-PE	2538	84.70%	
Weight status categories			
Underweight	585	19.50%	
Normal	1973	65.90%	
Overweight and obese	438	14.60%	

**Table 2:** Comparison of the results of MVPA, LPA, SB and PL by gender.

	Boy N = 829	CV	Girl N = 2167	CV	t	*p*	95% CI	
MVPA	3846.52 ± 2989.42	0.777	1960.02 ± 1998.65	1.020	16.791	0.000	1666.053	2106.948
LPA	1879.14 ± 1310.79	0.698	1597.71 ± 1269.61	0.795	5.303	0.000	177.335	385.538
SB	365.82 ± 174.96	0.478	415.00 ± 157.68	0.380	−7.071	0.000	−62.835	−35.541
PL	31.72 ± 6.79	0.214	29.22 ± 5.36	0.184	9.523	0.000	1.986	3.016

**Table 3:** Differences between groups with different weight status categories.

	Underweight N = 585	Normal N = 1973	Overweight and Obese N = 438	F	*p*
MVPA	2022.09 ± 2044.64	2628.55 ± 2535.4	2436.21 ± 2576.44	13.87	0.00
LPA	1609.63 ± 1276.23	1699.9 ± 1285.87	1654.1 ± 1306.39	1.18	0.31
SB	409.83 ± 151.35	397.16 ± 166.4	409.19 ± 169.58	1.92	0.15
PL	29.61 ± 5.12	30.14 ± 6.02	29.33 ± 6.29	4.37	0.01

**Table 4:** Correlation Analysis of variables (*n* = 2996).

	1	2	3	4
1. MVPA	/			
2. LPA	0.299 **	/		
3. SB	0.192 **	0.001	/	
4. PL	0.316 **	0.141 **	−0.124 **	/

** *p* < 0.01.

**Table 5:** Regression analysis of variable relationship.

	Effect	F	*p*	β	t	*p*	95% CI	
Model 1								
MVPA = cPL + e1	c	331.195	0.000	0.316	18.199	0.000	117.583	145.980
SB = a1PL + e2	a1	47.128	0.000	−0.124	−6.865	0.000	−4.450	−2.473
LPA = a2PL + e2	a2	60.456	0.000	0.141	7.775	0.000	22.946	38.422
MVPA = c’PL + b1SB + b2LPA + e3	c’	235.269	0.000	0.259	15.452	0.000	94.323	121.740
	b1			−0.160	−9.661	0.000	−2.893	−1.917
	b2			0.263	15.805	0.000	0.440	0.565
Model 2								
MVPA = cPL + e1	c	294.687	0.000	0.200	12.453	0.000	70.384	96.692
SB = a1PL + e2	a1	28.232	0.000	−0.085	−4.561	0.000	−3.377	−1.346
LPA = a2PL + e2	a2	39.531	0.000	0.097	5.257	0.000	13.291	29.103
MVPA = c’PL + b1SB + b2LPA + e3	c’	243.725	0.000	0.172	10.971	0.000	58.993	84.669
	b1			−0.104	−6.808	0.000	−2.012	−1.112
	b2			0.198	12.834	0.000	0.320	0.436

Note: model 2 adjusts gender, grade and major.

**Table 6:** Parallel mediation effect test.

	Effect	Boot SE	t	*p*	LLCI	ULCI	c’ cs	Percent Effect
Total effect of X on Y	83.538	6.709	12.453	0.000	70.384	96.692	0.200	
Direct effect of X on Y	71.831	6.548	10.971	0.000	58.993	84.669	0.172	85.986%
Indirect effect(s) of X on Y	Effect	Boot SE	Boot LLCI	Boot ULCI				
TOTAL	11.707	1.926	8.174	15.644				14.014%
SB	3.690	1.046	1.843	5.941				4.417%
LPA	8.017	1.672	4.879	11.380				9.597%

## Data Availability

The data presented in this study are available on request to qualified researchers from the corresponding author.
